# Phase angle, muscle tissue, and resistance training

**DOI:** 10.1007/s11154-023-09791-8

**Published:** 2023-02-10

**Authors:** Luís B. Sardinha, Gil B. Rosa

**Affiliations:** Exercise and Health Laboratory, Faculdade de Motricidade Humana, CIPER, Universidade de Lisboa, , Cruz Quebrada, Portugal

**Keywords:** Bioelectrical impedance analysis, Cellular Health, Muscle function, Phase angle, Resistance training

## Abstract

The biophysical response of the human body to electric current is widely appreciated as a barometer of fluid distribution and cell function. From distinct raw bioelectrical impedance (BIA) variables assessed in the field of body composition, phase angle (PhA) has been repeatedly indicated as a functional marker of the cell’s health and mass. Although resistance training (RT) programs have demonstrated to be effective to improve PhA, with varying degrees of change depending on other raw BIA variables, there is still limited research explaining the biological mechanisms behind these changes. Here, we aim to provide the rationale for the responsiveness of PhA determinants to RT, as well as to summarize all available evidence addressing the effect of varied RT programs on PhA of different age groups. Available data led us to conclude that RT modulates the cell volume by increasing the levels of intracellular glycogen and water, thus triggering structural and functional changes in different cell organelles. These alterations lead, respectively, to shifts in the resistive path of the electric current (resistance, R) and capacitive properties of the human body (reactance, Xc), which ultimately impact PhA, considering that it is the angular transformation of the ratio between Xc and R. Evidence drawn from experimental research suggests that RT is highly effective for enhancing PhA, especially when adopting high-intensity, volume, and duration RT programs combining other types of exercise. Still, additional research exploring the effects of RT on whole-body and regional BIA variables of alternative population groups is recommended for further knowledge development.

## Biological tissue function

Biological tissues are complex heterogeneous media composed of cells with distinguished intracellular characteristics and signaling systems that are encoded by genes in the nucleus of the cells and activated upon external signals from non-original cells or non-cellular components present in the human tissue [1]. Together, the interaction between the intracellular components (e.g., organelles structure and function, hydration levels) and extracellular matrices that provide structural and biochemical support to cells constitutes the basis of the development and function of body tissues, and more complexly, organs and systems. Although dynamic cellular processes are constantly ongoing to ensure a biological balance between tissue and organ function, mechanical and non-mechanical events may occur where this function is corrupted or altered [2]. While the exposure of the cell to extreme stress through pressure, abrasion, and rapid agitation, represents the most relevant mechanical factor influencing cellular health and tissue function, other parameters of a more physical, chemical, and biological dimension should be considered [1, 2]. Due to the direct influence of such alterations on cell structure and physiologic function, acting as a proxy of overall health, a deeper understanding of cell proprieties for cellular health monitoring purposes has been a target of current research.

Therefore, we begin this review by briefly highlighting different electric-based methodologies used to assess the general biophysical and bioelectric characteristics of cells in the field of body composition, health, and other domains. Then, we focus our attention on several biophysical parameters deriving from the response of the biological tissue to electric current flow, by addressing the concepts of impedance (Z), resistance (R), reactance (Xc), and phase angle (PhA). With a special interest in PhA, we further examine the potential of using this non-invasive marker to characterize cellular health. Next, a deeper analysis of the main biological and non-biological determinants of PhA and how they change in response to distinct types of resistance training (RT) is provided. Finally, we systematize and discuss the current evidence regarding the effect of RT programs on PhA, R, and Xc, and add future recommendations that should be taken into consideration when designing and implementing training programs.

## Biological tissue measurement

Since the early 1900s, biophysical characteristics of cells and tissues have highlight important markers of cellular integrity and function [3]. A prerequisite for appropriate cell function and the basic building block of living tissue is that it contains and is surrounded by a liquid electrolyte solution [4]. Under these conditions, both the endogenous and exogenous sources of electrical current (i.e., bioelectrical field), which respectively arise from the ionic activities inside the body cells (e.g., nerve cells) and derive from an external electrical excitation source (e.g., bioelectrical impedance device), are possible to measure. Endogenous sources of current have been widely used as a clinical marker to investigate the electric phenomenon of basic life processes, such as the somatic and autonomic nervous system response in different organs [5, 6].

When looking at other exogenous sources, which are of main interest in the bioimpedance field, special attention has been given to the concept of immitance of biological materials, which is an attribute combining both properties of impedance (i.e., ability to oppose the current flow) and admittance (i.e., ability to admit current flow) of biological materials. While covering the duality of admittance and impedance, known to be both frequency and geometry dependent properties, this parameter of immitance can now be measured using two- to eight-electrode systems to explain most of the dielectric behavior at tissue and cellular levels. One of the most common examples of the applicability of immittance consists of its use as a dependent variable in initially proposed Cole mathematical equations (i.e., equations often used to describe dielectric properties of the tissue). On the borderline between medical and nonmedical applications, the assessment of impedance and admittance proprieties has been particularly important given the possibility to monitor intra- and extracellular fluid indices as important determinants of health and muscle function, as well as to indirectly estimate other parameters of body composition (i.e., fat mass, fat-free mass) through predictive equations that include the model error to estimate total body water (TBW) and a specific assumption that fat-free mass is comprised of water [7, 8]. Beyond the extensive number of predicting equations allowing the estimation of whole-body tissues from the raw biophysical parameters and other variables such as weight, height, and age [9–11], there is now a growing research interest in understanding the implications of both impedance and admittance proprieties of different body regions (i.e., arms, legs, trunk), as these tend to differ in their shapes and biological constitutions [12]. Several equations predicting regional proportions of body composition have therefore been recently developed for healthy populations [9, 13–15], as well as for other special populations (e.g., athletes) [16], which has opened the possibility to further characterize tissue composition of each segment and determine limb asymmetries at both functional and structural levels.

## In vivo biological tissue measurement

The assessment of the electrical properties of the biological tissue assumes that the electric current flows passively and at different rates through the body depending upon its composition [17]. Within the field of body composition, investigating electrical proprieties at the cellular level mostly relies on surface electrode-based approaches (e.g., metal plates, suction, flexible and floating electrodes) to monitor exogenous conduction of applied alternate current (i.e., the amount of electric stimulation flowing in the tissue), and levels of voltage (i.e., the difference in electric potential between two points in an electric circuit). From the ratio between the maximum level of voltage and current, it is possible to determine the total opposition that a circuit, or part of it, offers to a direct or alternate applied current (i.e., electrical impedance, Z) [11, 18]. When looking at alternating current circuits, which are more complex and form the basis of the human body, the concept of Z involves not only R but also the property of reactance (X, in Ω), which is a measure or function of capacitance and frequency [19, 20]. Capacitance, for instance, represents an aggregate measure of the frequency-dependent responses of the cell membrane to briefly store electric current as it passes through the body tissues. Due to the presence of the capacitive elements that help to store electrical energy in the form of an electric field in the human body, the overall X may be classified as capacitive X (Xc). Depending on the relative contribution of Xc and considering the resistive (R) characteristics of the medium where the electric current flows, it is possible to determine the degree level that current leads or lags voltage in an alternating circuit, also known as phase angle (PhA, Φ) [18]. By geometrically quantifying the angular transformation of the ratio between Xc and R, researchers from the field of physics, engineering, and, more recently health, have been using PhA as an indicator to quantify the relative contribution of raw BIA parameters of different biomaterials.

From a physical point of view, PhA is considered a relative measure of the viscoelastic properties of a material, ranging from 0° (full resistive) to 90° (complete capacitive) [21]. Conceptually, PhA is negatively associated with R and positively associated with Xc, with this being true in any material with mixed viscoelastic properties. In the human body, for example, PhA typically ranges from 1º to 12º, with the R and Xc contributions varying according to a set of biological factors that will be later discussed. Although the biological meaning of PhA is not fully understood, this raw BIA parameter has been used as a non-invasive nutritional and health assessment tool to characterize the cellular health and intra- and extracellular fluids [22], which are, respectively, the major biological determinants of Xc and R in the human body [18]. The available literature suggests that lower PhA, consistent of reduced Xc, are attributable to reduced body cell mass (BCM) and to compromised selective permeability function of the cell, which are, in turn, related to lower levels of muscle strength, quality of life, and increased rates of hospitalization and mortality [23, 24]. On the other hand, higher levels of PhA are conceptually associated with increased levels of BCM, which is mainly composed of skeletal muscle mass (SMM), and directly linked to an improved cell membrane health [22]. With researchers having this information at hand, a growing body of evidence has been demonstrating a close and positive effect of sports performance, physical activity, and general health on PhA [25, 26].

## Determinants of phase angle

With a special interest in further understanding how PhA changes, a variety of investigations have suggested that BIA-derived parameters are determined by numerous factors ranging from simple cell characteristics to the tissue structural organization [27], which are expected to change with age, sex, and disease-specific parameters [7]. Thus, by adopting a biophysical point of view regarding how electric current flows throughout the body giving rise to the physiological responses previously identified, we will provide information on how cell structure and function, as well as body geometry and volumes, determines PhA.

### Determinants of phase angle—cell structure and function

**Fig. 1:**
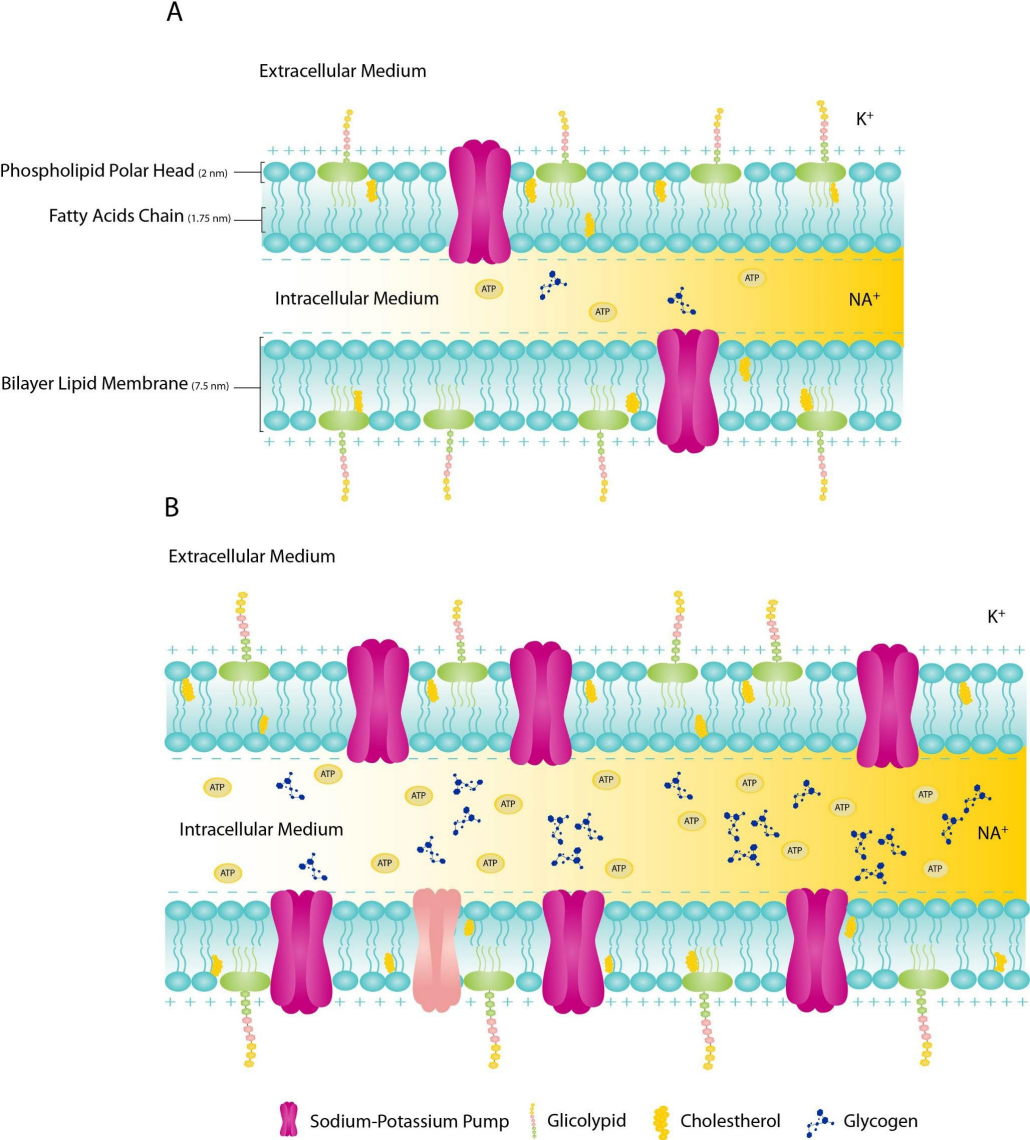
Bilayer lipid membrane structure without resistance training (A) and with resistance training-induced changes (B)

Beyond the extrinsic electrical properties that define how electric current flows through the tissue, greater attention has been given to the individual contribution of biological structures and functions at the cellular level (Fig. 1). One of the most important structures of the cell is its bilayer lipid membrane (BLM), whose biophysical function can be interpreted in terms of capacitance for energy and metabolism or signaling transduction [28, 29]. Evidence from late as the 1930 to the early 1950’s firstly suggested that when the electric current is carried through the membrane two consecutive bioelectrical processes occur [30, 31]. Since the BLM constitutes an insulating barrier between the electrically conductive solutions of the intra- and extracellular mediums, with the fatty acid chains representing the dielectric component, this cell structure has been referred to behave as a capacitor [32]. According to Hodgkin et al. [31], the BLM has a capacitance level of approximately 1 microfarad(µF)/cm^2^ and a permittivity constant of 8.85 × 10 − 8 µF/cm, which is mainly determined by the presence of heterogeneous lipidic elements (i.e., polar heads of the phospholipids and lipid rafts), acting as determinants of cell fluidity and important sources of energy storage [33]. Within an optimal range of frequency that is externally increased, the electric current reaches the cell membrane, increasing the electric charge accumulation at the lipidic components, expressing the level of Xc [33]. If the electric current flow increases towards the infinite (i.e., Cole models), the effect of Xc is gradually lost, with the externally applied current only flowing through the intra- and extracellular resistive path of the biological tissues [21].

Changes in cell membrane fluidity play a key role in the regulation of structural, functional, and dynamic properties of the membranes. Beyond the implications of exercise training on cell membrane function and structure, which will be posteriorly discussed in this review, a great debate exists about the main pathological mechanisms that underlie cell membrane disruption and contribute to cell death, recognized as one of six hallmarks of cancer [34]. In the early ’70s, the first homeoviscous adaptations including shifts in the mobility of cell membrane protein receptors and reduction in the degree of fluidity of membrane lipids were identified in cell structures of mammals with impaired tumor cells [35, 36]. With alterations in the saturation degree of the membrane phospholipids and cholesterol, the level of fluidity in the tumor cell membrane decreases, thus leading to malignant transformations (i.e., cell proliferation, differentiation, apoptosis) [37, 38]. The magnitude of these effects is specific to the type, stage, and sensitivity status related to each cancer [39]. Since changes in the cell membrane structure are expected to impact the biophysical properties of this organelle, markers of cell quantity and quality (e.g., PhA and Xc) have been recurrently used to discriminate between the biological characteristics of both healthy individuals and patients with a diagnosis of specific cancers [24] including advanced colorectal [40], lung [41], pancreatic [42], and breast cancer [43].

Another bioelectrical process taking place at the cell membrane considers the cell potential model, where important ion pumps, such as the sodium-potassium pump, are coupled in the cell membrane. In human excitable cells, the intracellular medium actively conserves a polarized potential of excitation between − 3 mV to -90 mV [44], with this process depending on the selective permeability of the cell membrane to ions [45], charged molecules embedded in cell membrane structure [46] and osmotic pressure equilibrium [47]. However, when an external electrical stimulation within an optimal range of the frequencies (e.g., kHz) previously proposed [21] builds up across the cell membrane, a passive process, consisting of the electrochemical ion exchanges between the intra and extracellular mediums through ion-specific leak channels (e.g., K^+^ leak channels), occurs below the depolarization cell membrane threshold potential [31]. If the threshold of excitation is reached, the ion’s selective channels open and the ions migrate according to the electrochemical gradient, which instantly reverses the negative charge of the intracellular medium up to + 30 mV [31]. It is through this process that electric current flows inside the intracellular medium with high electrolytic conductivity, and enables the measurement of the intracellular R, reflecting the level of intracellular water (ICW). Given the dynamic nature in which the various cellular structures act within these biological processes and influence the BIA-derived parameters of R, Xc, and consequently PhA, further research investigating the impact of distinct conditions (e.g., stress condition, physical exercise, or illness) is needed.

#### Determinants of phase angle—cell structure and function response to resistance training

**Fig. 2:**
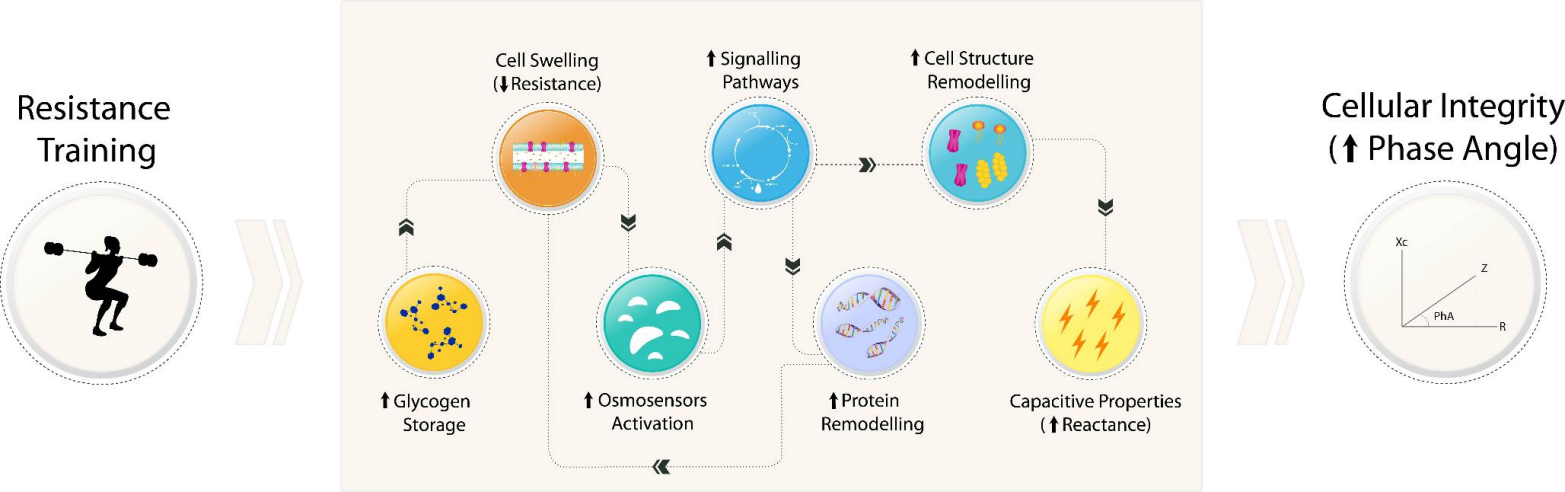
Physiological scheme representing the effect of resistance-based training on bioelectrical impedance-derived phase angle

From a biophysical perspective, understanding how exercise training modulates the cell structure characteristics and alters their function leads us to the following research questions—“What are and where do structural changes of cells occur in response to specific types of exercise, in particular RT? What are the implications of these changes on the determinants of PhA? As presented in Fig. 2, one important mechanism involved in structural changes at the cellular level following RT is linked to the increase of ICW (i.e., hydration-mediated cell swelling) [48]. Even though a decrease in predicted ICW (i.e., dehydration-mediated cell shrinking; -2.5%) is expected immediately after the completion of multiple sets of fatiguing resistance exercise [49], recent evidence suggests opposite adaptations with muscle swelling events occurring immediately after an initial session of RT [50]. Hirono et al. [50], for example, firstly demonstrated that the greater the muscle swelling immediately after an initial session of RT (3 sets of 8 reps at 80% 1 M), the greater the muscle hypertrophy after a 6-week (3 days/week) RT intervention with similar characteristics. While considering RT as a strong contributor to acute and cumulative muscle changes (i.e., muscle cell swelling) [51], previous studies addressing the long-term effects of RT programs have also suggested intracellular adaptations [52–59]. Following a 16-week RT program consisting of 3 days/week of 9 to 11 exercises with 3 sets of 8 to 12 RM, the authors found that the level of predicted ICW determined using a spectral BIA device increased by around + 9.5% in individuals of both sexes [52]. Similar positive trends ranging from + 3 to + 8.4% were observed in other investigations employing RT protocols in athletes [53] and healthy populations [54–59], with the extension of these findings being dependent upon the population characteristics, exercise type, and intensity level of training.

Within the field of physiology, it has been theorized that RT directly contributes to the phenomena of cell swelling induced by biological mechanisms related to hydrostatic and oncotic pressures occurring in the intracellular medium [60]. Contrary to the effects of pathological cell swelling conditions (e.g., cytotoxic edema), in which the reversible effects of cell swelling can become irreversible and progress to cell death, the response to RT is expected to be adaptive and completely reversible with the restoration of the normal function and structure of the cells [61]. One commonly suggested mechanism to explain cell swelling events relies on the susceptibility of glycogen, which is stored in the muscle tissue bound to water molecules in a proportion of 1:3 g [62], to change according to diverse types of exercise stimuli and muscle fiber stimulation [63]. With special attention to RT, although a rapid depletion of the available glycogen occurs during and immediately after an intensity-specific exertion, particularly in non-oxidative fibers [64], evidence suggests that the proportion of glycogen tends to increase to levels above rest concentrations, resulting in a marked increase in the order of approximately 4 g per 100 g of muscle (i.e., baseline ratio of ∼1.5 g of glycogen/100 g of muscle) [65]. This physiological adaptation, therefore, allows for the recruitment of glycolytic fibers during heavy RT for a longer sustained time, even though there is always a concomitant increase in lactate, growth hormone and reactive oxygen species acting as the key contributors to osmotic changes in muscle cells (i.e., increase in phosphocreatine and hydrogen ions) [66, 67]. While recognizing that the molecular weights of both lactate and hydrogen ions are smaller than that of muscle glycogen, the increase in both ion concentrations in response to RT is expected to provide an additional mechanism that accelerates the cell swelling according to the cell permeability gradients [66]. Thus, the higher the training volumes and intensities of RT programs (i.e., increased metabolic stress), the higher the rates of muscle swelling, and, consequently, the greater the hypertrophy.

Beyond other signaling processes explaining how glycogen is resynthesized and how lactate regulation processes occur, which are already available in literature review articles [68, 69], we speculate that the increased intracellular hydrostatic pressure resulting from increased glycogen availability particularly following carbohydrate loading favors cell hydration and swelling, thus contributing directly to a decrease in intracellular R. While adopting a deeper theoretical perspective, additional research using a spectral BIA device (i.e., prediction equations) demonstrated that the rise in glycogen availability after 72-hours of carbohydrate loading led to an increase in the predicted ICW (+ 3.4%), but not in ECW, of the specific limb group used for glycogen depletion [70]. According to the authors, this segment-specific increase in ICW (i.e., a proxy of the cell volume of the corresponding limbs) contributed not only to reduced intracellular R by -3.3%, but also to positively changing the whole-body ICW (+ 1.6%) and the overall level of TBW measured using deuterium dilution techniques (+ 2.4%) [70]. With these effects having a major impact on both regional and whole-body intracellular R levels, we cannot exclude the possibility of the results changing according to the characteristics of the limb-specific exercise used for glycogen depletion, the type of post-exercise food loading, and the time that elapsed between the exercise session and posterior assessment moments [70, 71].

When looking at the medium to long-term effect of RT on ICW, ECW, and TBW, the available data from 8-to 16-week RT programs clearly supports an increase in TBW measured using prediction models, particularly in the ICW component [54, 56–58, 72–75]. Despite this biological variable—ICW—being the key determinant of intracellular R, to the best of our knowledge, no intervention has been conducted to examine the impact of a RT program on intra- and extracellular R, separately. Instead, most studies focused their attention on the negative relationship between spectral BIA predictions of ICW and/or TBW, and total R (i.e., sum of intra and extracellular R), having shown that, in response to RT, the level of ICW and TBW increases approximately + 3.1% to + 10.5% and + 0.7% to + 7.5%, respectively, and the total R decreases in the order of -1.5% to -5.4% [54, 56, 58, 72–75]. Following the increase in ICW in response to RT, which is illustrated in Fig. 2B, the dynamic balance between intracellular protein synthesis and breakdown may be affected by molecular transduced signaling pathways (e.g., calcium-dependent pathways), whereby the intracellular protein balance shifts to favor synthesis over degradation [51]. Due to the increased hydrostatic pressure against the BLM, the cell initiates signaling responses, via integrins associated with the osmo-sensing process, reinforcing the membrane structure and assisting the growth of capacitive elements (i.e., phospholipids; Fig. 2B) [51]. Even if these changes do not affect the cell membrane thickness, which was shown to remain stable in response to RT (i.e., between 7 and 8 nm tick) [19], the available evidence sustains that these changes heavily contribute to expanding the cell membrane surface area and, consequently, to the increase in the overall level of capacitance [76]. By expanding intracellular proteins, the oncotic pressures inside the cell increase, resulting in passive movements of the ECW to the intracellular medium, which influences the overall electric resistive path and leads to an increasing level of capacitance. This phenomenon was recently proved to be true in adults and older adults, with evidence showing that a moderate-intensity 24-week RT, rather than a low-intensity RT of equal length, had the potential to improve two major factors derived from cell swelling events, i.e., the intracellular R index (+ 1.1%) and the level of capacitance (+ 6.2%) [77]. Although no information regarding changes in ICW or ECW components following the 24-week RT was made available, the authors reported that both capacitance (r = 0.42) and intracellular R index (r = 0.40) were positively associated with the changes in the thigh muscle CSA [77], thus allowing us to speculate that the hypertrophy processes has a positive impact on the overall level of capacitance. Since the intervention intensity and duration appear to be contributing factors for muscle tissue remodeling, which, in turn, relates to the level of capacitance improvement [77, 78], more research addressing this and other RT characteristics (i.e., type, volume, study population) is necessary.

One of the main responses to RT consists of increases in the BCM, known to be the most metabolically active component of the body and one of the major determinants of resting energy expenditure [79]. Due to the high costs and the restricted availability of using reference methods to assess BCM (e.g., whole-body ^40^ K counting), alternative methods using BIA derived parameters have emerged. With robust evidence showing a positive relationship of BCM with both total body capacitance [80] and parallel Xc models [81], there is now an ongoing debate on how changes in these BIA variables can predict changes in BCM following a RT program. Recent studies demonstrated that RT leads to increased BCM of both adults [59] and older adults [55]. However, no information is available regarding the type or index of BIA variables used to predict this component, which compromises the plausibility of analyzing this variable and limits its interstudy-comparability. Future studies addressing the effects of RT are, therefore, strongly recommended to use validated methods to determine the BCM, i.e., total body capacitance and parallel Xc models, as well as to provide descriptive information about the adopted models.

Considering other capacitive elements integrated into the cell membrane, such as the membrane cholesterol, research adopting acute sessions of strenuous endurance exercise demonstrated that levels of cholesterol depletion can reach 80% [82], thus representing a physiological marker of cell damage (i.e., protein mis-sorting) and possibly leading to a reduction in the overall capacitance level. Even though information on the short-term effect of endurance training on this cell membrane component exists in animal models [83], with evidence reporting an acute exercise-induced reduction in membrane cholesterol content, no evidence exploring the impact of short- and long-term RT on membrane cholesterol structures of humans is currently available.

Regarding the concentration of sodium-potassium pumps exhibited in Fig. 2B, evidence suggests that RT increases the content of pumps in both diseased [84] and healthy populations [82, 85] by 15%. Also, considering the long-term upregulation effect of RT on the NA^+^, K^+^, and ATPase in muscle cells [82, 86], the combined effect of the increase on these cell membrane structures is expected to enhance cell depolarization and repolarization processes, thus increasing the muscle contractile performance, preserving muscle function, and inhibiting the accumulation of fatigue at the cellular level of the muscle tissue. Although the increased electrolytic fluidity arising from the increase in sodium-potassium pumps could also represent a decrease in extra- to intracellular R and favorably increase PhA, particularly when applying stimulation frequencies within the β-dispersions range [87], no information to date exists linking these effects to changes in bioelectrical components.

### Determinants of phase angle—body geometry and volumes

Beyond the important implications of the cellular structures on Xc, R, and PhA, other morphological factors (i.e., macrostructure level) including body volume and geometry have been identified to influence the manner that electric current flows throughout the human body. Although a simple relation exists between body volume and impedance parameters, with evidence suggesting that the R of a cylindrical object with known homogeneous conductive properties is proportional to its length and inversely proportional to its cross-sectional area (CSA), the nature of this relationship is even more complex in the human body, which does not consist of a uniform cylinder, nor does it have one tissue with constant conductivity [11]. Instead, the geometrical shape of the human is more closely related to a series of 5 cylinders (i.e., upper and lower limbs, and trunk) consisting of mixed conductive and resistive properties that depend upon the tissue microstructure, hydration status, and concentration of electrolytic ions [12].

By adopting this 5-cylinder model, several investigations using tetrapolar BIA approaches sought to investigate the relative contribution of distinct body segments’ resistivity on overall Z and R. In 1980, Settle et al. [88] demonstrated that the arm and leg segments accounted, respectively, for approximately 40% and 45% of the whole-body R, even though they contributed for only 30% of the body volume in the current path. Following this knowledge, Lukaski and Scheltinga [89] stated that not all the limb R, but rather the R of the shorter segments of each limb (i.e., forearm R and the lower leg R), which correspond to only around 1–2% of the whole-body fat-free mass and 1.5-3% of the body weight, are the main contributors of whole-body R (61%). By taking a deeper look into the reasons behind the high contribution of the forearm and lower leg to whole-body R, two major reasons explaining this contribution have been pointed out. First, due to the limited CSA of these body segments, their contribution to the overall level of R will have much more impact compared to wider body segments (e.g., trunk) [11, 18]. Since the forearm and the lower leg have a more approximate cylindrical geometry, the rationale behind the previous assumption follows the idea that the R of a cylindrical object is inversely proportional to its CSA.

Another factor contributing to the increased R of the forearm and lower leg is related to the tissue composition of these segments. Compared to other body segments that integrate the 5-cylinder model [12], the forearm and lower leg segments contain much higher percentages of bone (10–16%) [90], which is known to have a constant low conductivity. Considering both factors (i.e., CSA and tissue composition) and extending their impact to the other segments of the body, we speculate that changes in the geometry and shape of arms and legs, in particular those occurring in the forearm and lower leg segments, may result in substantial changes in the Z and R, and consequently PhA, at both the segmental and whole-body level without affecting the body volume extensively.

#### Determinants of phase angle—body geometry and volume response to resistance training

The effect of aerobic and strength exercise on muscle phenotype and architecture has been widely appreciated in healthy and diseased populations, with robust evidence showing that particularly RT leads to a triad of events chronologically organized into neuromuscular adaptations that result in higher levels of strength and increased muscle size. According to the dose-response curve that best describes how these components interact in response to RT, experimental research suggests that the initial adaptations within the neuromuscular system (e.g., SMM activation) are firstly determined by overall increases in neural activation, motor unit synchronization, and muscle recruitment [91]. Only when neural adaptations begin to plateau (i.e., 2 to 4 weeks after starting RT), the myofibrillar proteins start to slowly increase (i.e., hypertrophy) [92], thus contributing to changes in the CSA of the muscle and influencing the biological path through which the electric current flows. Along with the rise in the dimension of the myofibrillar proteins that occurs across all types of muscle fibers [93], additional swelling events arising from catabolic processes occurring in the muscle tissue following RT may also favor an increase in the muscle CSA. Although no literature exists regarding the independent contribution of both mechanisms on changes in BIA-derived parameters during the early stages of RT (i.e., 3 to 4 weeks), decreases in whole-body and segmental R might be expected to occur.

Considering the medium and long-term effects of RT on muscle volume, it has long been recognized that shifts in muscle phenotype are the result of complex combinations between the characteristics of strength training and internal factors that include age, gender, genetics, energy, and, more recently, levels of muscle responsiveness [92, 94]. An important aspect of muscle adaptation is that muscle groups that are frequently involved in daily life activities, such as the forearm and lower leg muscles (i.e., main contributors to whole-body R), are expected to be already in a medium-high training state, thus limiting their growth level in terms of CSA and strength [18]. For this reason, most of the experimental research in this field moved towards understanding the impact of training programs with distinctive characteristics (e.g., frequency, intensity, muscle actions) on the CSA of muscle groups more susceptible to change in response to exercise.

By taking a deeper look at the dose-response for muscle tissue development, particular attention has been given to the elbow flexor and knee extensor muscles, with the available literature, sustaining a favorable trend toward the impact of RT on these muscle complexes [92, 95, 96]. Although increases in RT volume are expected to produce greater gains in muscle hypertrophy in the abovementioned muscles [95], systematized data of experimental evidence suggests that the degree of increase in muscle CSA may differ between limbs and change according to the nature of the RT program [95], thus affecting the magnitude of change in phase-sensitive BIA measure of both R PhA. Training programs using dynamic external resistances, such as free weights and weight machines, are expected to have an average increase in the CSA of elbow flexors (0.20% increase per day) that is greater than in the quadriceps (0.12% increase per day) [92], with these values being maximized when adopting training programs comprising of at least three to four exercise sessions per week with 4 to 6 sets (~ 40–60 repetitions) of exercises with intensity over 60–75% of 1-RM [92]. Since the R parameter of each limb is inversely proportional to its CSA [11], we speculate that the proportion of change in this and other BIA components (i.e., PhA) will be greater the more the RT involves maximum rates of CSA increase. Although it is still not clear whether changes in the CSA of the muscle in response to different modes of RT lead to changes in BIA-derived parameters at both whole-body and regional levels, a recent investigation demonstrated that increases in the CSA of the thigh segment following 24 weeks of moderate-intensity RT (60% of 1-RM), but not 24 weeks of low-intensity RT (40% of 1-RM), were positively correlated with whole-body intracellular R index, PhA and Xc [77]. Despite the significant increase (4%) in the thigh CSA of individuals involved in the low-intensity RT, no differences were found for muscle qualitative parameters [77], thus confirming our previous speculation that higher training intensities are needed to change R and PhA. Nevertheless, more research is needed to understand at what point moderate-to-high intensity RT influences the CSA and BIA-derived parameters of other muscles complexes with high responsiveness to hypertrophic training, which can have important implications on how electric current flows through the body (e.g., elbow flexors and knee extensors).

## Resistance training and phase angle—current evidence

To further clarify the effect of distinct types of RT on the biological structures that are of most importance in the modulation process of PhA and other BIA-derived components (i.e., Xc and R), we provide a deeper look at the findings of the most updated evidence in this research field (Table 1). To the present date, twenty-six investigations (i.e., 1262 individuals) addressing the effect of RT on the previously mentioned BIA-derived parameters in adult and older adult populations, but not in children, were identified and summarized in Table 1. From these, we were able to differentiate three types of RT programs—traditional RT, RT combined with other physical fitness domains, and RT combined with diet/oral supplementation, with most using phase-sensitive BIA devices and reporting positive changes in the PhA (> 80% of studies), independently of whether it was determined from a single or multi-frequency BIA device.

**Table 1:** General characteristics of the twenty-six investigations addressing the effect of resistance training on raw bioelectrical impedance parameters

Author	Sample	Experimental Conditions	RT Program	Device	Results Mean ± SD	∆ Mean ± SD ^δ^
Barbosa, 2018	G1: 16 F: 56.1 ± 5.5 yrs G2: 14 F: 52.7 ± 4.4 yrs	G1: Resistance training + Isoflavone G2: Resistance training + Placebo	10 weeks x3, 7 exercises, 2 sets x 15 reps	Biodynamics®, model 450c, 50 kHz	G1 **R**: T0: 604.9 ± 38.7, T1: 609.6 ± 46.7 **Xc**: T0: 67.9 ± 18.6, T1: 72.4 ± 7.4 **PhA**: T0: 6.7 ± 0.7, T1: 6.7 ± 0.6 G2 **R**: T0: 604.0 ± 29.4, T1: 589.5 ± 43.9 **Xc**: T0: 76.7 ± 12.2, T1: 70.7 ± 9.1 **PhA**: T0: 7.2 ± 1.0, T1: 6.8 ± 0.5	G1 **R**: 4.7 ± 43.3 **Xc**: 4.5 ± 16.2 **PhA**: 0.0 ± 0.7 G2 **R**: -14.5 ± 38.7* **Xc**: -6.0 ± 11.0* **PhA**: -0.4 ± 0.9
Campa, 2018	G1: 15 F: 65.6 ± 5.2 yrs G2: 15 F: 66.5 ± 4.3 yrs	G1: Control G2: Suspension Resistance training	12 weeks x N/A, 6 exercises, 4 sets x 12 reps	Akern ®, BIA 101 Anniversary, 50 kHz	G1 **R**: T0: 536.2 ± 46.7, T1: 540.7 ± 46.2 **Xc**: T0: 52.3 ± 7.9, T1: 51.2 ± 7.2 **PhA**: T0: 5.6 ± 0.4, T1: 5.5 ± 0.5 G2 **R**: T0: 555.2 ± 46.9, T1: 540.2 ± 49.2 **Xc**: T0: 53.6 ± 4.1, T1: 57.1 ± 4.5 **PhA**: T0: 5.6 ± 0.4, T1: 5.9 ± 0.5	G1 **R**: 4.5 ± 46.5 **Xc**: -1.1 ± 7.6 **PhA**: -0.1 ± 0.5 G2 **R**: -15.0 ± 48.1* **Xc**: 3.5 ± 4.3* **PhA**: 0.3 ± 0.5*
Campa, 2021	G1: 11 M: ~67 yrs G2: 11 M: ~67 yrs G3: 11 M: ~67 yrs	G1: Suspension Resistance training G2: Traditional Strength training G3: Control	12 weeks x3, 7 exercises, 3 sets x 12 reps	Akern ®, BIA 101 Anniversary, 50 kHz	G1 **R/H**: T0: 285.9 ± 22.9, T1: 276.8 ± 21.6 **Xc/H**: T0: 32.3 ± 5.0, T1: 33.1 ± 5.1 **PhA**: T0: 6.5 ± 0.6, T1: 6.8 ± 0.7 G2 **R/H**: T0: 263.9 ± 35.5, T1: 258.0 ± 35.8 **Xc/H**: T0: 29.9 ± 3.9, T1: 30.3 ± 4.0 **PhA**: T0: 6.5 ± 0.7, T1: 6.8 ± 0.8 G3 **R/H**: T0: 274.8 ± 16.3, T1: 286.9 ± 10.5 **Xc/H**: T0: 29.4 ± 2.3, T1: 29.4 ± 2.1 **PhA**: T0: 6.1 ± 0.6, T1: 5.8 ± 0.4	G1 **R/H**: -9.1 ± 22.3* **Xc/H**: 0.8 ± 5.1 * **PhA**: 0.3 ± 0.7* G2 **R/H**: − 5.9 ± 35.7 **Xc/H**: 0.4 ± 3.95 **PhA**: 0.3 ± 0.8* G3 **R/H**: 12.1 ± 14.3 **Xc/H**: 0.0 ± 2.21 **PhA**: -0.3 ± 0.5*
Cunha, 2018	G1: 22 F: 68.0 ± 4.5 yrs G2: 20 F: 69.7 ± 6.0 yrs G3: 20 F: 68.2 ± 4.3 yrs	G1: Control G2: Low Intensity Resistance Training G3: High Intensity Resistance Training	12 weeks x3, 8 exercises, 1–3 sets x 10–15 reps	Xitron Hydra ®, model 4200, 1-1000 kHz	G1 **R**: T0: 589.1 ± 74.3, T1: 594.6 ± 71.1 **Xc**: T0: 58.7 ± 10.1, T1: 55.9 ± 8.6 **PhA**: T0: 5.7 ± 0.6, T1: 5.4 ± 0.6 G2 **R**: T0: 552.7 ± 51.8, T1: 53.10 ± 45.6 **Xc**: T0: 56.3 ± 5.3, T1: 59.5 ± 4.5 **PhA**: T0: 5.9 ± 0.6, T1: 6.1 ± 0.5 G3 **R**: T0: 603.5 ± 59.9, T1: 570.9 ± 58.6 **Xc**: T0: 58.1 ± 7.6, T1: 61.9 ± 8.5 **PhA**: T0: 5.5 ± 0.6, T1: 5.9 ± 0.6	G1 **R**: 5.5 ± 72.8 **Xc**: -2.8 ± 9.4 **PhA**: -0.3 ± 0.6* G2 **R**: -24.6 ± 49.0* **Xc**: 3.2 ± 5.0* **PhA**: 0.2 ± 0.5* G3 **R**: -32.6 ± 59.2* **Xc**: 3.8 ± 8.1* **PhA**: 0.4 ± 0.6*
Dos Santos, 2016	33 F: 68.7 ± 5.7 yrs	Resistance training	12 weeks x N/A, 8 exercises, 3 sets x 10–15 reps	Xitron Hydra ®, model 4200, 1-1000 kHz	**R**: T0: 531.7 ± 61.0, T1: 529.7 ± 57.1 **Xc**: T0: 54.4 ± 6.0, T1: 55.9 ± 4.7 **PhA**: T0: 5.9 ± 0.5, T1: 6.1 ± 0.5	**R**: -2.0 ± 59.2 **Xc**: 1.4 ± 4.9 **PhA**: 0.2 ± 0.5*
Dos Santos, 2020	G1: 18 F: >60 yrs G2: 19 F: >60 yrs G3: 18 F: >60 yrs	G1: Control G2: Resistance training with narrow zone G3: Resistance training with wider zone	8 weeks x3, 8 exercises, 3 sets x 5–15 reps	Xitron Hydra ®, model 4200, 1-1000 kHz	G1 **R**: T0: 572.1 ± 72.3, T1: 587.2 ± 75.7 **Xc**: T0: 54.7 ± 5.0, T1: 53.6 ± 7.0 **PhA**: T0: 5.6 ± 0.9, T1: 5.3 ± 0.6 G2 **R**: T0: 564.0 ± 44.8, T1: 555.4 ± 51.2 **Xc**: T0: 53.6 ± 5.1, T1: 55.7 ± 5.9 **PhA**: T0: 5.5 ± 0.7, T1: 5.8 ± 0.6 G3 **R**: T0: 574.5 ± 55.8, T1: 549.2 ± 60.5 **Xc**: T0: 54.5 ± 6.8, T1: 57.8 ± 5.4 **PhA**: T0: 5.5 ± 0.9, T1: 6.1 ± 0.7	G1 **R**: 15.1 ± 74.1* **Xc**: -1.1 ± 6.2 **PhA**: -0.3 ± 0.8* G2 **R**: -8.6 ± 48.3* **Xc**: 2.1 ± 5.5 **PhA**: 0.3 ± 0.7* G3 **R**: -25.3 ± 58.3* **Xc**: 3.3 ± 6.2* **PhA**: 0.6 ± 0.8*
Fukunda, 2016	20 F: 71.9 ± 6.9 yrs	Resistente training	3/6months weeks x3, 5 exercises, 1–3 sets x 8–12 reps	RJL Systems ®, Quantum II, 50 kHz	**R**: T0: 603.7 ± 53.4, T1: 604.0 ± 62.5, T2: 599.3 ± 69.2 **Xc**: T0: 50.6 ± 7.6, T1: 51.6 ± 7.0, T2: 52.9 ± 9.2 **PhA**: T0: 4.8 ± 0.6, T1: 4.9 ± 0.5, T2: 5.0 ± 0.7	**R**: 0.3 ± 58.5, -4.4 ± 62.8† **Xc**: 1.0 ± 7.3, 2.3 ± 8.5† **PhA**: 0.1 ± 0.6*, 0.2 ± 0.7*†
Gobbo, 2022	G1: 155 M: ~19 yrs G2: 115 M: ~19 yrs	G1: Physical training routine G2: Physical training routine + specific sport training	34 weeks x5, 8 exercises, 2 sets x 7–15 reps	RJL Systems ®, Quantum II, 50 kHz	G1 **R specific**: T0: 314.8 ± 27.6, T1: 312.7 ± 26.5 **Xc specific**: T0: 41.9 ± 5.5, T1: 45.7 ± 6.5 **PhA**: T0: 7.6 ± 0.8, T1: 8.3 ± 0.9 G2 **R specific**: T0: 311.7 ± 29.6, T1: 310.8 ± 28.3 **Xc specific**: T0: 40.1 ± 5.7, T1: 44.0 ± 6.1 **PhA**: T0: 7.3 ± 0.7, T1: 8.1 ± 0.8	G1 **R specific**: -2.1 ± 27.1^ N/A^ **Xc specific**: 3.8 ± 6.1^ N/A^ **PhA**: 0.8 ± 0.9^ N/A^- G2 **R specific**: -0.9 ± 29.0^ N/A^ **Xc specific**: 3.9 ± 5.9^ N/A^ **PhA**: 0.8 ± 0.8^ N/A^
Hernández-Jaña, 2021	G1: 11 F: 20.1 ± 1.9 yrs G2: 27 F: 18.9 ± 1.8 yrs	G1: Control G2: Resistance training + Cardiorespiratory training	12 weeks x5, 6–8 exercises, 3 sets x 10–12 reps	InBody ®, S100, 1 kHz, 5 kHz, 50 kHz, 250 kHz, 500 kHz, 1 MHz	G1 **Xc**: T0: 28.1 ± 1.8, T1: 28.2 ± 2.2 **PhA**: T0: 5.8 ± 0.4, T1: 5.7 ± 0.4 G2 **Xc**: T0: 26.2 ± 2.6, T1: 27.9 ± 2.7 **PhA**: T0: 5.7 ± 0.4, T1: 6.2 ± 0.5	G1 **Xc**: 0.1 ± 2.02 **PhA**: -0.1 ± 0.4 G2 **Xc**: 1.7 ± 2.68* **PhA**: 0.5 ± 0.5*
Langer, 2019	98 M: 18.8 ± 0.5 yrs	Resistance training + Calisthenics + Cardiorespiratory training + Circuit training + Swimming training	6 months x5, N/A	RJL Systems ®, Quantum II, 50 kHz	**R**: T0: 473.0 ± 39.6, T1: 461.8 ± 40.3 **Xc**: T0: 60.7 ± 6.0, T1: 64.5 ± 6.2 **PhA**: T0: 7.3 ± 0.7, T1: 8.0 ± 0.6	**R**: -11.2 ± 30.4* **Xc**: 3.8 ± 5.5* **PhA**: 0.7 ± 0.6*
Martin-Alemañy, 2016	G1: 11 F 19 M: ~30 yrs G2: 10 F 25 M: ~35 yrs	G1: Control/Oral supplementations G2: Oral supplementation + Resistance training	6/12 weeks x2, 4 exercises, 4 sets x 30 reps	RJL Systems ®, Quantum N/A, 50 kHz	G1 **R**: T0: 585.2, T1: 598.0, T2: 567.0 **Xc**: T0: 63.0, T1: 64.0, T2: 64.0 **PhA**: T0: 5.9, T1: 6.0, T2: 6.2 G2 **R**: T0: 572.6, T1: 553.0, T2: 538.0 **Xc**: T0: 57.8, T1: 58.0, T2: 57.7 **PhA**: T0: 5.8, T1: 5.9, T2: 6.1	G1 **R**: 12.8 ± N/A*, 18.2 ± N/A*† **Xc**: 1.0 ± N/A*, 1.0 ± N/A*† **PhA**: 0.2 ± 0.31*, 0.3 ± 0.6*† G2 **R**: -19.6 ± N/A*, 34.6 ± N/A*† **Xc**: 0.2 ± N/A*, -0.1 ± N/A*† **PhA**: 0.04 ± 0.5*, 0.33 ± 0.5*†
Nabuco, 2019	G1: 22 F: 67.5 ± 5.2 yrs G2: 21 F: 66.2 ± 9.4 yrs G3: 23 F: 66.5 ± 7.1 yrs	G1: Resistance training + Whey Protein-Placebo G2: Resistance training + Placebo-Whey Protein G3: Resistance training + Placebo-Placebo	8 + 12 weeks x3, 8 exercises, 3 sets x 8–12 reps	Xitron Hydra ®, model 4200, 1-1000 kHz	G1 **R**: T0: 573.7 ± 68.5, T1: 563.2 ± 61.2 **Xc**: T0: 53.5 ± 8.6, T1: 56.6 ± 8.8 **PhA**: T0: 5.3 ± 0.7, T1: 5.7 ± 0.7 G2 **R**: T0: 579.4 ± 85.1, T1: 567.3 ± 82.2 **Xc**: T0: 54.5 ± 9.4, T1: 58.2 ± 9.8 **PhA**: T0: 5.4 ± 0.6, T1: 5.8 ± 0.5 G3 **R**: T0: 620.2 ± 58.2, T1: 607.9 ± 61.1 **Xc**: T0: 57.6 ± 6.2, T1: 60.5 ± 5.6 **PhA**: T0: 5.3 ± 0.5, T1: 5.7 ± 0.5	G1 **R**: -10.5 ± 65.2 **Xc**: 3.1 ± 8.7* **PhA**: 0.4 ± 0.7* G2 **R**: -12.1 ± 84.0 **Xc**: 3.7 ± 9.6* **PhA**: 0.4 ± 0.6* G3 **R**: -22.3 ± 59.7 **Xc**: 2.9 ± 5.9* **PhA**: 0.4 ± 0.5*
Nunes, 2018	66 F: 68.8 ± 4.6 yrs	Resistance training	12 weeks x3, 8 exercises, N/A sets x 10–15 reps	Xitron Hydra ®, model 4200, 1-1000 kHz	**R**: T0: 579.0 ± 73.0, T1: 573.0 ± 73.6 **Xc**: T0: 54.2 ± 7.9, T1: 55.9 ± 8.0 **PhA**: T0: 5.4 ± 0.6, T1: 5.6 ± 0.6	**R**: -6.0 ± 73.3 **Xc**: 1.7 ± 8.0* **PhA**: 0.2 ± 0.6*
Olvera-Soto, 2019	G1: 39 M 7 F: 27.5–49.5 yrs G2: 32 M 16 F: 41.5–53.0 yrs	G1: Control G2: Resistance Training + Cholecalcipherol	12 weeks x3, 6 exercises, 3 sets x 8 reps	Seca ®, mBCA 515, 1-1000 kHz	G1 **R**: T0: 609.2 ± 97.9, T1: 606.5 ± 98.1 **Xc**: T0: 51.7 ± 14.2, T1: 52.1 ± 15.0 **PhA**: T0: 4.8 ± 0.9, T1: 4.8 ± 0.9 G2 **R**: T0: 577.7 ± 111.7, T1: 580.4 ± 131.4 **Xc**: T0: 47.9 ± 15, T1: 46.9 ± 16.9 **PhA**: T0: 4.6 ± 0.9, T1: 4.5 ± 0.9	G1 **R**: -2.7 ± 0.98 **Xc**: 0.4 ± 14.6 **PhA**: 0.0 ± 0.9 G2 **R**: 2.7 ± 122.7 **Xc**: -1.0 ± 16.0 **PhA**: -0.1 ± 0.9
Osco, 2021	G1: 19 F: 69.7 ± 8.2 yrs G2: 18 F: 70.1 ± 6.7 yrs	G1: Traditional Resistance training G2: Elastics Resistance training	12 weeks x3, 7–8 exercises, 2–3 sets x 8–15 reps	The Nutritional Solutions Corporation ®, BIA/Vitality Analyzer, 50 kHz	G1 **R/H**: T0: 402.8 ± 66.5, T1: 388.7 ± 10.1 **Xc/H**: T0: 32.0 ± 4.3, T1: 33.7 ± 5.1 **PhA**: T0: 4.8 ± 0.6, T1: 5.1 ± 0.9 G2 **R/H**: T0: 414.9 ± 70.7, T1: 401.2 ± 58.0 **Xc/H**: T0: 33.2 ± 2.9, T1: 32.2 ± 4.7 **PhA**: T0: 4.6 ± 0.6, T1: 4.6 ± 0.7	G1 **R/H**: -14.1 ± 62.1* **Xc/H**: 1.7 ± 4.8* **PhA**: 0.3 ± 0.8* G2 **R/H**: -13.7 ± 65.3 **Xc/H**: 1.0 ± 4.1 **PhA**: 0.0 ± 0.7
Otsuka, 2022	G1: 9 M 8 F: 63.5 ± 8.5 yrs G2: 8 M 8 F: 63.6 ± 8.1 yrs G3: 8 M 9 F: 63.5 ± 8.3 yrs	G1: Control G2: Low Intensity Resistance Training G3: Moderate Intensity Resistance Training	12/24 weeks x 3, 4 exercises, 3 sets x 14 reps	ImpediMed ®, model SFB7,1-1000 kHz	G1 **PhA**: T0: 6.1 ± 0.8, T1: 5.9 ± 0.7, T2: 6.1 ± 0.8 G2 **PhA**: T0: 6.5 ± 0.9, T1: 6.2 ± 0.9, T2: 6.6 ± 1.0 G3 **PhA**: T0: 6.3 ± 0.8, T1: 6.3 ± 0.9, T2: 6.5 ± 0.9	G1 **PhA**: -0.2 ± 0.3*, 0.0 ± 0.3 G2 **PhA**: -0.2 ± 0.3*, 0.1 ± 0.3 G3 **PhA**: 0.0 ± 0.3, 0.3 ± 0.3*
Ribeiro, 2016	G1: 28 M: 22.2 ± 4.3 yrs G2: 31 F: 23.2 ± 4.1 yrs	G1: Resistance training G2: Resistance training	8/16 weeks x3, 9–11 exercises, 3 sets x 8–12 reps	Xitron Hydra ®, model 4200, 1-1000 kHz	G1 **R**: T0: 515.2 ± 45.6, T1: 496.9 ± 37.2, T2: 490.7 ± 46.7 **Xc**: T0: 64.7 ± 5.0, T1: 64.6 ± 5.6, T2: 64.3 ± 5.3 **PhA**: T0: 7.2 ± 0.6, T1: 7.4 ± 0.6, T2: 7.5 ± 0.6 G2 **R**: T0: 625.5 ± 68.7, T1: 615.2 ± 75.9, T2: 601.6 ± 75.9 **Xc**: T0: 69.3 ± 8.4, T1: 70.4 ± 10.2, T2: 70.4 ± 9.3 **PhA**: T0: 6.3 ± 0.6, T1: 6.5 ± 0.7, T2: 6.7 ± 0.7	G1 **R**: -18.3 ± 42.0*, -24.5 ± 46.2*† **Xc**: -0.1 ± 5.3, -0.4 ± 5.16† **PhA**: 0.2 ± 0.6*, 0.3 ± 0.6*† G2 **R**: -10.3 ± 72.6*, -23.9 ± 72.6*† **Xc**: 1.1 ± 9.4, 1.1 ± 8.88† **PhA**: 0.2 ± 0.7*, 0.4 ± 0.7*†
Ribeiro, 2017a	G1: 17 F: 69.0 ± 5.1 yrs G2: 22 F: 69.1 ± 5.8 yrs	G1: Resistance training medium volume G2: Resistance training high volume	12 weeks x2/3, 8 exercises, 1 set x 10–15 reps	Xitron Hydra ®, model 4200, 1-1000 kHz	G1 **R**: T0: 507.6 ± 48.3, T1: 499.2 ± 64.8 **Xc**: T0: 53.6 ± 6.3, T1: 55.2 ± 4.1 **PhA**: T0: 6.1 ± 0.9, T1: 6.4 ± 0.9 G2 **R**: T0: 535.7 ± 59.8, T1: 518.6 ± 49.4 **Xc**: T0: 53.9 ± 5.5, T1: 57.6 ± 4.1 **PhA**: T0: 5.8 ± 1.0, T1: 6.4 ± 0.9	G1 **R**: -8.40 ± 58.3* **Xc**: 1.6 ± 5.5* **PhA**: 0.3 ± 0.9* G2 **R**: -17.1 ± 55.3* **Xc**: 3.7 ± 5.0* **PhA**: 0.6 ± 0.9*
Ribeiro, 2017b	G1: 25 F: 69.7 ± 6.6 yrs G2: 26 F: 68.9 ± 5.8 yrs G3: 25 F: 66.8 ± 4.2 yrs	G1: Constant load resistance training G2: Pyramid load resistance training G3: Control	8 weeks x3, 8 exercises, 3 sets x 8–12 reps	Xitron Hydra ®, model 4200, 1-1000 kHz	G1 **R**: T0: 586.5 ± 65.2, T1: 564.9 ± 74.2 **Xc**: T0: 55.6 ± 6.2, T1: 57.5 ± 7.6 **PhA**: T0: 5.6 ± 0.5, T1: 5.8 ± 0.6 G2 **R**: T0: 574.7 ± 59.5, T1: 559.8 ± 60.4 **Xc**: T0: 53.9 ± 7.7, T1: 57.9 ± 8.4 **PhA**: T0: 5.4 ± 0.7, T1: 5.6 ± 0.6 G3 **R**: T0: 580.5 ± 80.9, T1: 587.0 ± 79.2 **Xc**: T0: 55.7 ± 9.1, T1: 54.6 ± 8.0 **PhA**: T0: 5.6 ± 0.5, T1: 5.5 ± 0.5	G1 **R**: -21.6 ± 70.1* **Xc**: 1.9 ± 7.0* **PhA**: 0.2 ± 0.6* G2 **R**: -14.9 ± 60.0* **Xc**: 4.0 ± 8.0* **PhA**: 0.2 ± 0.7* G3 **R**: 6.5 ± 80.1 **Xc**: -1.1 ± 8.7 **PhA**: -0.1 ± 0.5
Skelton, 1995	G1: 20 F: 79.5 yrs G2: 20 F: 79.5 yrs	G1: High resistance training G2: Control	12 weeks x1, 8 exercises, 3 sets x 4–8 reps	RJL Systems ®, model 109, 50 kHz	G1 **PhA**: T0: 5.7 ± 0.7, T1: 6.2 ± 1.1 G2 **PhA**: T0: 6.0 ± 0.7, T1: 6.3 ± 1.1	G1 **PhA**: 0.5 ± 1.0* G2 **PhA**: 0.3 ± 0.7*
Souza, 2016	G1: 22 F: 67.1 ± 4.5 yrs G2: 19 F: 67.3 ± 4.3 yrs	G1: Control G2: Resistance training	12 weeks x3, 8 exercises, 3 sets x 10–15 reps	Xitron Hydra ®, model 4200, 1-1000 kHz	G1 **R**: T0: 585.2 ± 74.2, T1: 590.3 ± 74.3 **Xc**: T0: 57.6 ± 9.5, T1: 56.5 ± 8.3 **PhA**: T0: 5.6 ± 0.6, T1: 5.5 ± 0.6 G2 **R**: T0: 591.2 ± 75.0, T1: 572.1 ± 69.2 **Xc**: T0: 57.2 ± 8.2, T1: 58.8 ± 9.5 **PhA**: T0: 5.5 ± 0.5, T1: 5.9 ± 0.6	G1 **R**: 5.1 ± 74.3 **Xc**: -1.1 ± 9.0 **PhA**: -0.1 ± 0.6 G2 **R**: -19.1 ± 72.3* **Xc**: 1.6 ± 8.9 **PhA**: 0.4 ± 0.6*
Stratton, 2022	21 M: 21.9 ± 2.6 yrs	Lifestyle intervention with resistance training	6 weeks x3, 7 exercises, 3 sets x 8–12 reps	InBody ®, model 770, 1-1000 kHz Seca ®, mBCA 515/514,1-1000 kHz RJL Systems ®, Quantum V, 50 kHz	InBody 770 **R**: T0: 545.4 ± 51.9, T1: 520.3 ± 45.4 **Xc**: T0: 64.8 ± 7.4, T1: 63.1 ± 6.5 **PhA** T0: 6.8 ± 0.6, T1: 6.9 ± 0.5 Mbca 515 **R**: T0: 559.8 ± 53.1, T1: 528.8 ± 49.5 **Xc**: T0: 64.1 ± 7.3, T1: 62.1 ± 6.7 **PhA**: T0: 6.5 ± 0.5, T1: 6.7 ± 0.5 RJL Quantum V **R**: T0: 478.3 ± 49.5, T1: 450.7 ± 46.2 **Xc**: T0: 66.2 ± 7.5, T1: 63.5 ± 7.3 **PhA** T0: 7.9 ± 0.7, T1: 8.0 ± 0.7	InBody 770 **R**: −25.1 ± 23.4* **Xc**: −1.7 ± 4.0* **PhA**: 0.1 ± 0.2* mBCA 515 **R**: −31.0 ± 30.2* **Xc**: −2.0 ± 4.6* **PhA**: 0.2 ± 0.2* Quantum V **R**: −27.6 ± 21.0* **Xc**: −2.7 ± 4.1* **PhA**: 0.1 ± 0.3*
Tinsley, 2019	31 F: 22 ± 3 yrs	Resistance training + Protein supplementation	8 weeks x3, 5–6 exercises, 4 sets x 8–12 reps,	Seca ®, mBCA 515/514, 1-1000 kHz	**R**: T0: 695.0 ± 80.0, T1: 679.0 ± 68.0 **Xc**: T0: 69.0 ± 8.0, T1: 70.2 ± 7.8 **PhA**: T0: 5.7 ± 0.5, T1: 5.9 ± 0.6	**R**: -16.0 ± 74.7* **Xc**: 1.2 ± 7.9 **PhA**: 0.2 ± 0.6*
Tomeleri, 2018	G1: 24 F: 71.0 ± 5.4 yrs G2: 22 F: 68.8 ± 4.6 yrs	G1: Resistance training G2: Control	12 weeks x3, 8 exercises, 3 sets x 10–15 reps	Xitron Hydra ®, model 4200, 1-1000 kHz	G1 **R**: T0: 560.3 ± 56.1, T1: 547.1 ± 56.7 **Xc**: T0: 53.3 ± 7.9, T1: 55.9 ± 8.7 **PhA**: T0: 5.4 ± 0.6, T1: 5.8 ± 0.7 G2 **R**: T0: 579.8 ± 71.5, T1: 584.1 ± 70.3 **Xc**: T0: 57.0 ± 9.8, T1: 55.4 ± 8.3 **PhA**: T0: 5.6 ± 0.5, T1: 5.4 ± 0.5	G1 **R**: -13.2 ± 56.4* **Xc**: 2.6 ± 8.3* **PhA**: 0.4 ± 0.7* G2 **R**: 4.3 ± 70.9 **Xc**: -1.6 ± 9.1 **PhA**: -0.2 ± 0.5
Toselli, 2020	G1: 21 F: 53.7 ± 9.3 yrs G2: 21 F: 58.7 ± 8.5 yrs	G1: High resistance training G2: Low resistance training	24 weeks x1-3, 7 exercises, 4 sets x 8–12 reps	Akern ®, BIA 101 Anniversary, 50 kHz	G1 **R/H**: T0: 299.1 ± 28.4, T1: 295.6 ± 28.8 **Xc/H**: T0: 31.3 ± 3.0, T1: 33.7 ± 2.0 **PhA**: T0: 6.0 ± 0.5, T1: 6.5 ± 0.5 G2 **R/H**: T0: 303.3 ± 32.4, T1: 309.8 ± 33.8 **Xc/H**: T0: 33.9 ± 6.6, T1: 33.8 ± 6.2 **PhA**: T0: 6.2 ± 0.8, T1: 6.4 ± 0.6	G1 **R/H**: -3.5 ± 28.6 **Xc/H**: 2.4 ± 2.7* **PhA**: 0.5 ± 0.5* G2 **R/H**: 6.5 ± 33.1 **Xc/H**: -0.1 ± 6.4 **PhA**: 0.2 ± 0.7
Zanelli, 2015	G1: 7 M: ~23 yrs G2: 7 M: ~23 yrs	G1: Resistance Training + Supplementation (Non-trained) G2: Resistance Training + Supplementation (Trained)	7/28 days x3, 4 exercises, 3 sets x 10–12 reps	Biodynamics®, model 310, 50 kHz	G1 **PhA**: 7.3 ± 0.7, T1: 7.6 ± 0.8, T2: 7.8 ± 0.6 G2 **PhA**: T0: 8.4 ± 0.8, T1: 8.3 ± 0.7, T2: 8.5 ± 0.9	G1 **PhA**: 0.3 ± 0.8*, 0.5 ± 0.6† G2 **PhA**: -0.1 ± 0.8, 0.2 ± 0.9†

Abbreviations: G, experimental group, H, height, N/A, not available, PhA, phase angle, R, resistance, RT, resistance training, SD, standard deviation, Xc, reactance

δAbsolute mean and standard deviation values of change (∆ Mean ± SD) in phase angle, resistance, and reactance

*Difference between baseline (T0) and moment 1 (T1), and baseline (T0) and moment 2 (T2) at the 0.05 level

†Comparison between baseline (T0) and moment 2 (T2)

**Fig. 3:**
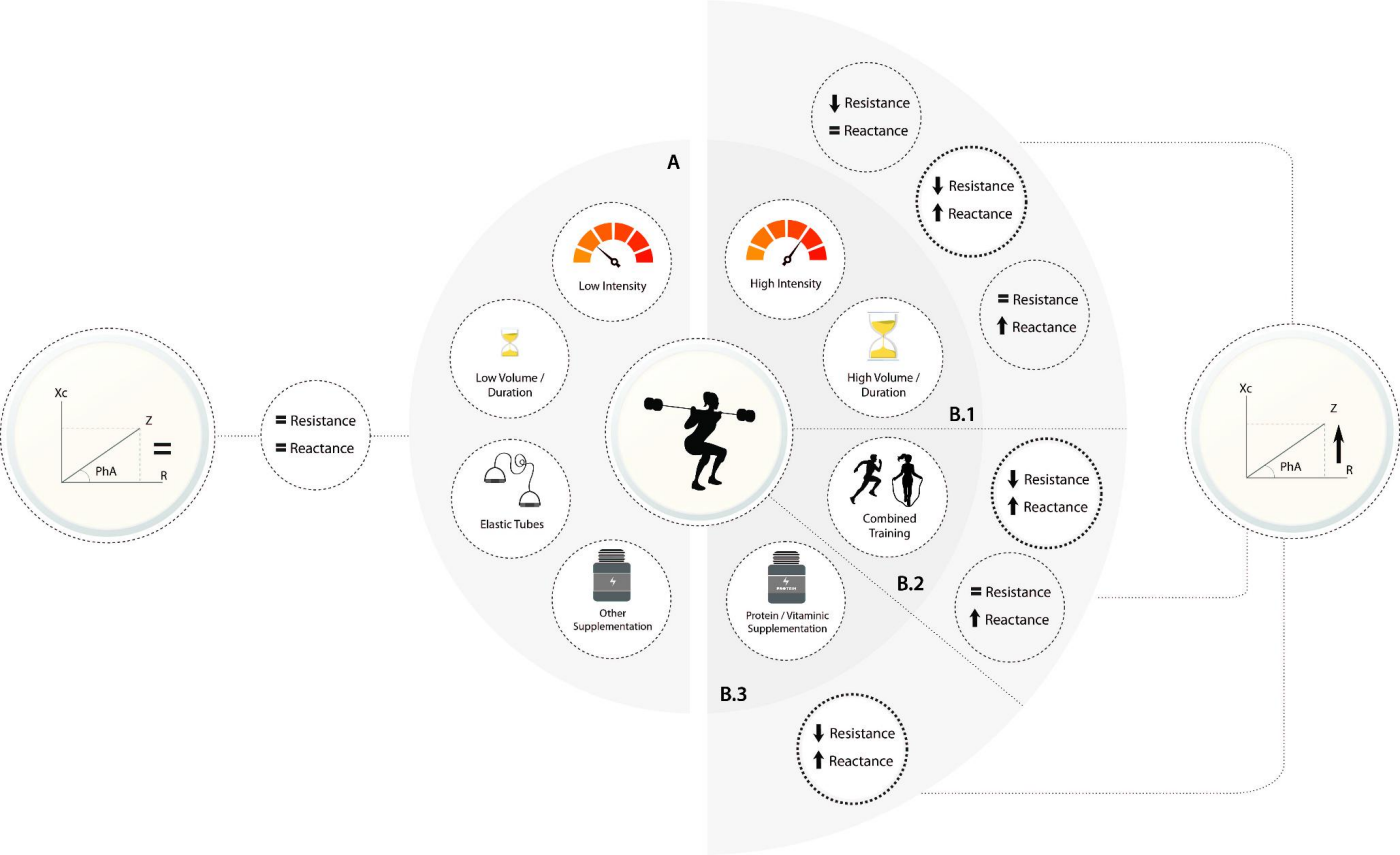
Bioelectrical impedance-derived phase angle response to low-intensity, low-volume/duration, elastic tubing, and non-protein supplementation (A), high intensity and high volume/duration (B.1), combined training (B.2), and protein/vitamin supplementation (B.3)

While being the first to explore the concept of traditional RT on whole-body PhA, Skelton et al. [97] did not find changes in this raw BIA parameter in women aged 75 and older after 12 weeks of low-intensity strength training (3 sets of 4 to 8 repetitions). Although the authors reported increases in the isometric strength of both upper and lower limbs, no changes were found in the arm muscle circumference (i.e., a proxy of lean body mass) [97]. As represented in Fig. 3A, the lack of change in the PhA following a low-intensity level of RT may be due to the well-known relationship between the CSA of the upper limbs, here represented by the arm muscle circumference, and the whole-body measure of R, which were expected to remain constant in this investigation. Similar to what has been previously reported in older women [55, 97], no changes in PhA, R, and Xc were found in two recent studies addressing the effect of a 24-week low-intensity (40% of 1-RM) and low-volume (1 day/week RT) RT program on muscle quality indicators in middle-aged adults [77, 98]. Despite none of these investigations reporting a substantial increase in levels of strength, which contrasts with evidence from the first experimental research study [97], the low-intensity RT program was found to improve muscle growth of the thigh segment. Interestingly, the authors reported that changes in muscle volume were not accompanied by alterations in the whole-body R [77], even though a decrease in the R of this segment would be expected. In the absence of changes in the biological markers of cell membrane integrity and cellular hydration (Xc and R, respectively), and consequently PhA in response to low-intensity RT, which are illustrated in Fig. 3A, the next research steps must evolve not only towards the implementation of training programs with higher intensities and volumes, but also towards a deeper understanding of the impact of low-intensity RT on BIA-derived parameters at the segmental level, which is an important marker of muscle quality in older adults.

With the decline in SMM quantity around 3 to 8% per decade after the age of 30 and even higher rates during older adulthood [99], changes in the metabolic, functional, and structural organization of the muscles occur, affecting the qualitative nature of this biological tissue. Considering the beneficial effect of medium to high-intensity RT programs to counteract age-related changes [100], we provide a specific age-group review of the effect of RT programs on raw BIA parameters, with a special interest in PhA. In adults, to our best knowledge, only two investigations examined the changes in PhA following a traditional hypertrophy-oriented RT. Different from what has been found from low-intensity programs, we have previously shown that a 16-week hypertrophy-oriented RT had a significant time effect on the PhA in younger adults, with PhA increasing approximately + 1.7% (men, R: -3.6%, Xc: -0.2%) and 3.2% (women, R: -1.7%, Xc: 1.6%) in the first 8 weeks of training, and + 5% (men, PhA: +4.3%, R: -4.8, Xc: -0.6; women, PhA: +5.8% R: -3.8%, Xc: 1.6%) in the following 8 weeks [72]. Although no changes in the Xc component were identified, suggesting that RT does not influence cellularity, cell size, and integrity of the cell membrane, we found that 8 and 16-week programs may be effective to increase the intracellular hydration and SMM, thus contributing to decrease R and increase PhA [72].

A recent investigation has attempted to address this issue in the female adult population while implementing a 24-week RT program with two training volumes (1 vs. 3 days/week) [98]. Although no changes in phase-sensitive BIA-derived PhA, Xc, and R of the low-volume group training were identified, the authors reported significant increases in PhA (+ 8.3%) and Xc index (+ 7.7%) in the high-volume training group [98], which could be attributed to the reduction in the ECW/ICW and consequent reinforcement of the cellular membrane (Fig. 3B1). Even though these findings indicate that 8 to 16-week RT favors the increase in PhA through the decrease in R (higher contribution of hydration), whereas 24-week programs with high training volume improve PhA by increasing Xc (higher contribution of BCM, for example), further research adopting control groups is needed to confirm these findings and thus allow for a more consistent analysis of the impact of RT on PhA.

When looking at the impact of RT on BIA properties of older adult populations, the most up-to-date evidence suggests positive effects of exercise programs on PhA (Table 1). Two systematic reviews with meta-analysis [101, 102] have recently addressed this issue, providing valid evidence that distinct RT programs affect PhA depending on the combined or independent effect of increasing Xc and/or decreasing R (Fig. 3B1). By following the previous analysis model adopted by Campa et al. [101], and only considering RT programs without the influence of other exercise types and diet/supplementation, we identified eleven intervention studies exploring the effect of RT on PhA in light of changes in other BIA-derived components (i.e., Xc and R). Although PhA was found to increase by about + 1.9 to + 10.6% in all 8 to 24-week RT programs, with the largest improvements being observed in response to RT of greater duration and/or intensity, the relative contribution of Xc and R did not follow the same trend, thus allowing us to identify three profiles, as presented on Fig. 3B1.

Regarding the most prevalent profile of change in raw BIA parameters following RT programs, we identified five investigations with similar training volumes that resulted in higher PhA (+ 2.9 to + 10.3%) [55, 73, 74, 103, 104]. According to the authors, the improvements in whole-body PhA following a conventional RT program were determined from the combined decrease in R (-5.4% to -3.2%) or R/H (-3.5% to -3.2%) and increase in Xc (+ 6.6% to + 7.4%) or Xc/H (+ 2.5% to + 5.3%) emerging from the physiological process explained in the proposed schematic model of Fig. 2. Despite these significant effects, attention must be given to understanding whether the RT programs using alternative methodologies and equipment influenced PhA, Xc, and R. Similar changes in both BIA-derived variables were recently identified among RT programs adopting conventional weight training and suspension-based approaches [55, 73, 74, 103, 104], however, the results from the elastic tubing exercises did not follow the same trend [55], thereby suggesting that this training approach did not provide enough intensity to exert changes on cell hydration indicators, nor in cell membrane structures that determine biophysical parameters of the muscle tissue (Fig. 3A). Therefore, even though there is an increase in overall strength resulting from RT protocols with distinctive characteristics, which is particularly important during older adulthood, these findings suggest that reaching elevated levels of RT may be a key element in promoting adaptations in terms of cell health.

Beyond the simultaneous contribution of both R and Xc as markers of cellular health and BCM, other investigations have highlighted that the decrease observed in R occurred without any compensatory change in Xc, as well as the opposite, with this having an impact on PhA. A previous investigation demonstrated that a 12-week RT program produced significant long-term increases in PhA (+ 6.5%) in older women, with these findings being sustained by the sole change in the R component (-3.2%), accompanied by changes in the level of ICW [56]. By following our proposed schematic model representing the overall mechano-physiological pathway to increase PhA (Fig. 2), several conclusions can be drawn from this research, the most apparent being that a significant increase in cell hydration, resulting from higher glycogen availability or increased oncotic pressures at the cellular level, led to decreases in both intracellular and overall. We also speculate that the level of ICW may not have been able to trigger signaling mechanisms that would later stimulate protein synthesis and cell membrane reinforcement causing the cellular capacitance to increase. Similar findings addressing this issue were recently published [58], with the authors indicating that 8 weeks of crescent pyramid-based training performed with narrow repetition zones were not effective to simultaneously improving the PhA and Xc of older women (PhA: +5.3%, R: -1.5%). As an alternative, the authors suggest that performing a wider range of repetitions may maximize the metabolic response during the initial sets and the mechanical effects in the latter sets [58], thereby heightening anabolism and enhancing the capacitance properties of the cell membrane [51]. Since additional benefits in terms of cell structure remodeling are expected when performing RT programs and achieving high levels of intensity (pyramid-based training with a wide range) seem feasible, it, therefore, makes sense to promote high-intensity RT for older adults as a means to foster changes in cellular health.

An additional profile related to exercise-induced changes in PhA via a rise in the Xc component, but not R, can be further identified [54, 57, 105]. While examining the effects of a 12-week RT program on 66 resistance-trained older women, the authors demonstrated that exercise only had an impact on Xc (+ 6.8%) and PhA (+ 7.5%) [57]. Although no information was provided regarding the level of intracellular hydration, which would facilitate the understanding of the lack of change in R following exercise, we speculate that due to the previous training experience of these women, baseline ICW was already high, likely as a result of rises in intracellular glycogen levels, thereby lowering the magnitude for improvement. Since Xc is only expected to increase after cell swelling events, as presented in Fig. 2, one possible explanation for these findings may include that the effects of cell structure remodeling may persist longer than cell swelling events, whereby frequent changes occur in response to exercise.

Despite the three types of biophysical response profiles identified here, the evidence is clear regarding the implications of RT on PhA. For instance, Martins et al. [102] suggested that strategies to improve PhA must consider not only the type of RT and equipment used, but also the periodization, intensity, and volumes of the training stimulus (Fig. 3B1). More specifically, the largest effect sizes on PhA are most commonly observed among RT programs with 12 weeks with 3-week sessions, consisting of 3 to 4 sets with 12 repetitions, with load adjustment when the upper limit of the repetition interval is reached [102]. These effects are not only dependent on the level of previous training of the participants but are also influenced by the inclusion of other types of exercise (e.g., cardiorespiratory training) or diet/supplementation programs (Fig. 3B2 and B3).

## Resistance training with combined intervention and phase angle—current evidence

Beyond purely RT, which demonstrated to be effective for increasing cell mass and health, recent studies highlighted concurrent training (i.e., resistance and cardiorespiratory training) and multicomponent-based training (i.e., resistance, cardiorespiratory, calisthenics, and swimming training) as the most appropriate alternatives to enhance whole-body and segmental PhA (+ 8.7 to + 11%) [59, 106, 107]. As shown in Fig. 3B2, combining RT with cardiorespiratory training (5 days/week; 30 min/session) may increase whole-body and segmental PhA (+ 9.1%) [59] following the concomitant decrease in fat mass and increase in lean soft tissue mass, with the later known to be a strong determinant of intracellular hydration. Similar levels of PhA increase (+ 8.7%), resulting from the simultaneous decrease in R (-2.4%) and increase in Xc (+ 6.3%) levels, were also found following a multicomponent-based training [107] with the authors highlighting an increase in lean soft tissue mass and consequent increase in the overall content of water. By knowing that increased amounts of ICW and ECW diminish R values, and consequently increase PhA, there is a positive perspective of adopting prolonged training protocols to enhance cellular health, particularly during adulthood.

Despite the undeniable potential of using this type of training to improve body composition and muscle quality markers, caution must be taken when discussing these findings. Since concurrent training or multicomponent training programs tend to involve higher training volumes, compared with traditional RT programs, the improvement in PhA may not only be explained by the type of training adopted, but also by the principles of duration and volume previously addressed and further illustrated in Fig. 3B1. Also, considering age as a major determinant of PhA [27], the interpretation of findings regarding this type of training should be restricted to the study population (i.e., young adult population). As an alternative, future studies are recommended to further investigate the real potential of using this training type on the biophysical response of populations with different characteristics, as well as to examine the long-term effect of training programs with less duration and lower volumes.

## Resistance training with supplementation and phase angle—current evidence

Although there is extensive research suggesting a strong influence of supplementation on parameters of muscle quantity and quality, few studies exist addressing the combined effect of RT and different types of supplementation on PhA. General improvements in PhA (+ 3.5%), as well as in R (-2.3%), in response to 8-week RT with protein supplementation (25 g whey protein) have been recently reported among the adult population [108]. With the rate of improvement in PhA being slightly higher than previously reported in a similar investigation (+ 3.4%) [109], the additional protein supplementation, mediated by increased SMM, added greater benefits in terms of PhA and R, than the conventional RT program [108] (Fig. 3B3). Since the effect sizes of this methodological approach on changes in muscle quality and quality may be affected, not only by the type of supplementation used but also by other exercise-related parameters (e.g., training experience) that strongly influence muscle recruitment and muscle growth, further research is needed to look at the impact of different types of protein supplementation combined with RT on BIA-derived parameters.

Few investigations exist that demonstrate the potential effect of combining RT with specific diets and other types of supplements to improve PhA. A recent study investigating the longitudinal agreement between different BIA devices in response to a 6-week RT program with a high caloric diet reported slight levels of improvement in PhA (+ 1.8 to + 2.6%) and R (-4.5 to -5.8%), but not Xc [110] between the devices. Similarly in a 12-week RT program combined with oral supplementation high in folate and vitamin B6, positive effects on PhA (+ 1.7%) and Xc (+ 1.6%) were demonstrated after 6 and 12 weeks of training among adults [111]. Although the RT volume in this trial was relatively small when compared to other studies, these findings suggested a positive effect of adding vitamin supplementation on muscle quality markers (Fig. 3B3). These findings may, however, not be extensible to other types of supplementation (e.g., isoflavone), since the effect of such methodological conditions was not investigated or proved to not be effective to enhance PhA and other qualitative parameters [111] (Fig. 3A). Therefore, understanding how the RT with distinct characteristics may have increased effects when combined with diverse types of supplementation may be of particular interest, especially in physically impaired populations.

## Technical remarks of phase angle measurement

Despite the undeniable effect of distinct types of RT on the biological structures that are of most importance to modulate PhA, caution must be taken when comparing data from studies using different devices (e.g., phase-sensitive single frequency and multifrequency BIA) and accessory equipment (i.e., electrodes). Even though previous results suggested that different BIA devices should not be used interchangeably [112], mostly because non-phase-sensitivity methodologies are not free of latent model error prediction on PhA calculation [8], data from the present review suggests similar increases in PhA (phase-sensitive single frequency: +1.3% to + 8.7% and multifrequency: +1.5% to + 10.6%) following different types of RT.

Another factor accounting for variability in the PhA measurement concerns the type of electrodes used, with growing evidence reporting the use of silver-silver chloride (Ag/AgCl) electrodes. Although using these Ag/AgCl electrodes with low intrinsic Z and low skin contact impedance is recommended to minimize measurement error, few studies have reported the type of electrodes used [113]. From the twenty-six investigations considered in this review, only 3 studies using multifrequency (+ 1.5 to + 5% PhA increase) reported the type of electrode used (dual-tab pre-gelled Ag/AgCl electrodes) [77, 110, 114]. To standardize the BIA assessment methodologies and to further allow more valid comparisons between findings from different studies, researchers are recommended to always use the specific electrodes for each equipment and to follow the same BIA assessment protocol.

Finally, although the majority of studies included in this review reported relatively low measurement errors for PhA (technical error of measurement: 0.1 to 0.3; standard error of measurement: 0.13 to 0.21), these values coincide with the expected absolute difference in PhA following a traditional low to medium intensity/volume RT program (PhA change of 0.04 to 0.3), thus limiting the interpretation of the significance of our findings. Even though this is not true for high intensity/volume or combined RT programs, where the expected changes in PhA (0.4 and 0.7) exceed the expected measurement errors, findings from the present review suggest that caution must be taken when drawing conclusions about the effect of low to medium intensity/volume RT on PhA. To further improve the quality of the results and allow establishing concrete relationships between RT and PhA changes, researchers are encouraged to reduce both technical and standard errors of measurement that mostly occur during pretest preparation procedures (e.g., skin cleaning and electrode placing), and report measurement errors whenever possible.

## Future recommendations

There are still some challenges that need to be addressed to further improve our understanding of how changes in R and Xc can be improved through RT programs. First, there is a need to extend RT programs to children and adolescents and other underexplored adult populations (e.g., unhealthy individuals) following the recommendations of the recently available evidence [101, 102]. Moreover, future investigations adopting RT programs should also consider adopting different types of strength training (e.g., training to failure), diet/supplementation (e.g., high-protein diet), and combination of different exercise types, to further understand their implications at the cellular level, and their consequent contributions to changes in muscle quality markers. Particularly in individuals with sarcopenia or involved in weight loss programs, where physiological alterations (i.e., hydration, BCM, body mass) lead to changes in BIA qualitative parameters, monitoring the effects of distinct RT programs must be of prime importance. Along with the analysis of raw data (PhA, R, and Xc), special attention should be paid to bioelectrical impedance vectors, which are less prone to interpretation errors [115]. In fact, the vectorial approach appears to be more efficient, as it combines two types of influential variables (raw BIA parameters and vector positioning), which provide information concerning the changes in total body water and extracellular/intracellular water ratio (ECW/ICW) and the variations in the absolute amount of the BCM [11]. As this is recognized as a more efficient methodology, future studies should consider including this vectorial approach when exploring the effect of RT on body composition.

Due to the increased availability of octopolar BIA devices able to measure the PhA of each segment individually, researchers should also aim to investigate how segmental BIA-derived parameters relate with changes in CSA of muscle complexes that have high responsiveness to RT and have important implications on how electric current flows through the body. It should be emphasized that different devices also have different performances in the raw variables that impact the calculation of the PhA, as well as different sensitivities, which determines the need for practitioners, researchers, and clinicians to consider the need for equipment-specific reference values. Beyond the expected changes in the raw BIA parameters following RT, an increase in the SMM and BCM, i.e., metabolically active components of the body, is foreseeable. Therefore, we strongly recommend researchers to provide descriptive details (e.g., development model, predicting variables or models—e.g., total body capacitance/parallel Xc model, changes in fluid components—e.g., ICW/ECW) about the prediction models used to predict the previously mentioned components. Finally, we strongly recommend researchers to consider the inclusion of a control group, report all raw BIA parameters, and include a follow-up period to test whether the RT effects may persist in the long term.

## Abbreviations

BIA: Bioelectrical impedance analysis
BLM: Bilayer lipid membrane
BCM: Body cell mass
CSA: Cross-sectional area
ICW: Intracellular water
ECW: Extracellular water
PhA: Phase angle
R: Resistance
RM: One-repetition maximum
RT: Resistance training
SMM: Skeletal muscle mass
TBW: Total body water
Xc: Reactance
Z: Impedance
